# The circle of security parenting and parental conflict: a single case study

**DOI:** 10.3389/fpsyg.2014.00887

**Published:** 2014-08-12

**Authors:** Chiara Pazzagli, Loredana Laghezza, Francesca Manaresi, Claudia Mazzeschi, Bert Powell

**Affiliations:** ^1^Department of Philosophy, Social and Human Sciences and Education, University of PerugiaPerugia, Italy; ^2^Astrea (Association for Therapy and Research in Developmental and Adult Psychopathology)Rome, Italy; ^3^Circle of Security InternationalSpokane, USA

**Keywords:** attachment based intervention, support to parenting, single case study, secure base, pre-school age, parental conflict, circle of security parenting

## Abstract

The Circle of Security Parenting (COS-P) is an early attachment based intervention that can be used with groups, dyads, and individuals. Created in the USA and now used in many countries, COS-P is a visually based approach that demonstrates its central principles through videos of parent/child interactions. The core purpose of the COS-P is to provide an opportunity for caregivers to reflect on their child's needs and on the challenges each parent faces in meeting those needs. Even though there is a wide range of clinical settings in which child/parent attachment is an important component of assessment there is limited empirical data on when and how attachment based interventions are appropriate for specific clinical profiles and contexts. The aim of this paper is to present a clinical application of COS-P in order to explore and reflect on some specific therapeutic tasks where it works and on some clinical indicators and contexts appropriate for its application. A single case study of a father, “M.” (43 years old) in conflict for the custody of his 5 years old daughter is reported. The Adult Attachment Projective Picture System (AAP), the Parenting Stress Index, the Strengths and Difficulties Questionnaire, and the Parental Alliance Measure, were administered pre- and post-intervention. The clinical significance analysis method revealed that numerous changes occurred in the father. The AAP showed improvements in the level of agency of self. M. made gains in his capacity to use internal resources and to increase his agency of self. M. was classified as recovered in his perception of the child's functioning and as improved in his parenting stress and parenting alliance with the mother. Considerations on specific contexts and clinical indicators for the application of COS-P are proposed.

## Introduction

Numerous systematic intervention programs driven by attachment theory and research have been developed and early programs often involve problematic or at-risk parents-child relationships and are aimed to shift the developmental pathway in a more adaptive way (Van IJzendoorn et al., 1995; Zeanah et al., 2011). Parent's internal working models (IWMs), parenting behaviors, and the intervention process as an engine of therapeutic change are the three therapeutic tasks of attachment based interventions as defined by Berlin et al. (2008). The Circle of Security (COS; Marvin et al., 2002) intervention, and the Circle of Security Parenting (COS-P; Cooper et al., 2009) are directly derived from attachment theory and research, and are based on these three therapeutic tasks (Berlin et al., 2008). The COS intervention and the COS-P, have the same underlying model based on Ainsworth's idea of a Secure Base and a Haven of Safety (Ainsworth et al., 1978). The focus of both interventions is on helping parents develop reflective capacity regarding internal processes that drive problematic parent/child interactions as well as develop a coherent understanding of their child's attachment needs in order to break insecure parenting patterns.

In this article, we first discuss COS intervention characteristics in order to introduce the more recent COS-P, and then its clinical application through a single case study. The therapeutic tasks highlighted by Berlin et al. (2008) are used to identify the areas to be investigated by the use of a performance-based tool and self-reports. Finally, some clinical indications and contexts appropriate for COS-P application are discussed.

The COS intervention is an evidenced based intervention that joins psycho-educational, cognitive-behavioral, psychodynamic understanding and intervention techniques (Ramsauer et al., 2014). COS has been manualized as a time-limited group psychotherapy for parents of young children (aged 1–5). Intervention protocol is designed to promote: attachment security in early parent/child relationships through supporting and strengthening the caregiver's skills regarding perceptions and understanding of the child's needs; observational and inferential skills; reflective functioning; emotion regulation, and empathy for the distress that the caregiver's unregulated emotions cause in their children (Cooper et al., 2005; Powell et al., 2013). Increasing parental awareness of their child's exploratory and attachment needs helps the parent reflect and learn how their automatic maladaptive responses to those needs creates a problematic self-perpetuating feedback loop. That loop feeds painful unregulated parental affect that can lead to the triggering of their child's insecure attachment strategies (Marvin et al., 2002; Hoffman et al., 2006; Powell et al., 2009).

A central element of COS intervention is the use of multiple graphics and videos. The COS graphic is a map that allows parents to follow their child's relationship needs, helping the parent become more emotionally available to them. Using animated graphics, the alternating child's attachment and exploration needs in a “secure base” relationship are figuratively illustrated as a “circle of security.”

Parents are invited to identify and reflect on the specific child's needs within the activation of the exploratory system, and within the activation of the attachment system, together with providing a supportive presence to respond to their child's needs (Page and Cain, 2009). Videotapes are used extensively to promote parental reflection on parent/child interactions. Review of selected video clips of parent/child interaction allows immediate feedback that enhances the parents' awareness of how interactive behaviors affects their child's responses. Highlighting positive moments in the parent/child interactions is used to facilitate the engagement of difficult-to-reach caregivers (McDonough, 2000, 2004).

In order to tailor the intervention for each dyad's needs (caregiver/child) a preliminary standardized assessment procedure is used to identify both the strengths and struggles in the dyadic relationship. A parent perception interview is used (Circle of Security Interview) to understand the parent's IWMs (Bowlby, 1973) that includes positive and negative child representations and a clinical model to organize treatment based on core defensive relationship strategies used by each parent. The assessment is also based on a systematic observation of parent/child interaction (Ainsworth et al., 1978; Cooper et al., 1999). Edited clips from the preliminary systematic observation of parent/child interaction are used to “individualize” videos of the participating dyads that will be shown during group intervention.

Relatively new is the creation of the COS-P which is a parent-education program offering the core components of COS intervention protocol (Cooper et al., 2009). COS-P occurs in eight sessions that join education about attachment with an opportunity for caregivers to reflect on their child's needs and the challenges each parent faces in meeting those needs (Zeanah et al., 2011). COS-P aims to implement decades of attachment research in an accessible step-by-step process for use in group settings, home visitation, or individual counseling.

COS-P is implemented in eight sessions (Cooper et al., 2009). In the first sessions, the parent is introduced via animated graphics and video clips to the “Circle of Security” roadmap illustrating the child (and adult's) alternating needs for attachment and exploration, together with the need for a supportive parental presence. The parent is accompanied to individualized their child's attachment needs behind the child behavior, and the profound effect of experiences in which child's needs are met (“Being-With”) or unmet (“Being-Without”) in the caregiver relationship. In the central part of the program there is a shift from the child to the caregiver where caregiver proper functioning is depicted as being always bigger, stronger, wiser, and kind, take care of the child's needs when possible, and take charge when necessary. Limited circles are then introduced, both the insecure, where the caregiver is able to meet only the attachment or exploration needs of the child, and the disorganized circle, where parent is frightening/frightened being mean, weak or gone, i.e., mentally absent. After this the parent is encouraged to reflect on his own needs and vulnerabilities with the aid of music (i.e., “Shark Music”), illustrating how unregulated and perceived dangerous affects influences and shapes caregiving. When the completion of the limited circles is more possible, then parental reflective function on unregulated emotional responses to the child's behavior is enhanced. The parent is guided in a new way to stay with the child's needs as a solution to relationship struggles, and the experience of disruption and repair acquires a central role in the sessions. Lastly, key relationship challenges are reviewed and positive changes are highly remarked.

COS-P is designed to be more scalable and less intense than COS intervention. With COS-P preliminary standardized assessment is not performed, video is not tailored to each dyad's specific needs, and the number of weeks in which it takes place is less. Rather than “individualized” videos of the participating dyads and of the responses from the pre-intervention interview, COS-P uses DVDs of archived videotapes that are selected as prototypical examples of parent/child interactions. Those DVDs contain videos of secure and problematic parent/child interactions, healthy caregiving options and are used in conjunction with attachment related animated graphics designed to clarify the central principles of the intervention.

The COS-P has the advantage of being more flexible in terms of timing, practicality and more adaptable for community-based use while the COS intervention requires a significant investment in terms of space, video equipment, and preparation time. Hoffman et al. (2006) pointed out the need for research on the efficacy of COS-P and on the intervention on only one parent (or couple) at a time.

The present paper aimed to show a clinical application of COS-P in order to reflect on some specific therapeutic tasks where it works and on some contexts and clinical indicators appropriate for its application. The case study presented involves a father in conflict for the custody of the 5 years old daughter. This case study was chosen because it involved a high level of parental conflict for child custody which is considered one of the four clinical contexts in which attachment perspective should have a central role (Zeanah et al., 2011) and because it might have been able to demonstrate change in long-term unresolved parental conflict.

With a pre/post-intervention design, a performance-based tool and self-report questionnaires were administered to the father. According to Berlin's et al. (2008) therapeutic tasks of attachment based interventions, parent's IWMs and parenting behavior were chosen to be assessed. Possible changes in parenting alliance were also assessed. The co-parenting relationship has been identified as having significant effects on parent/child relationship (Gable et al., 1994; McHale and Rasmussen, 1998; Frosch et al., 2000). Research has shown significant associations between higher parenting alliance and better parenting, less parenting stress and fewer attachment relationship difficulties with the child (Cohen and Weissman, 1984; Frank et al., 1991).

This study was designed to explore the presence of improvements into the following: (a) parental IWMs, particularly the underlying functioning dimensions of IWMs via a performance-based attachment tool; (b) parenting behaviors measured via a self-report that assessed parenting stress in child rearing. A self-report regarding the child was also administered to evaluate changes in parental perception of child's behavioral adjustment, and positive and negative parent attributions of the child's characteristics; (c) parenting alliance measured with a self-report.

## Materials and methods

### Case presentation

M., a 43-year-old male, arrived at a private psychotherapist requesting help to improve his parenting capacities with his 5 years old daughter. His main goal was to demonstrate that he was a good father and to obtain the daughter's custody having a chronically unresolved conflict with the child's mother. M. lived with the mother of his daughter for one year and because of their high conflict they decided to not marry. He recounts that he didn't want children but that after his daughter's birth he became very involved in her caretaking. M. reported that the child's mother didn't want him to care their daughter and he was deeply convinced that the child's mother only wanted money from him. He described his daughter as difficult to manage and having severe behavioral and emotional problems such as concentration difficulties, impulsivity and nervousness. M. appeared highly distressed by the quality of his relationship with his daughter, but responsibility for all his daughter's difficulties were attributed exclusively to her mother or to specific intrinsic characteristics of the child. He showed low empathy and awareness of his child's needs and low reflective capacity regarding his own internal processes.

When M. started treatment, the situation was characterized by high levels of parental conflict for child custody as he and the child's mother each had an attorney and the level of antagonism and aggressiveness between the parents was increasing. The request to improve his parenting capacities seemed to be moved mainly by the conflict with the child's mother. M. seemed more motivated by the desire for revenge against the mother than to find new ways to connect with his daughter. The COS-P program was offered within the context of individual therapy with the aim of helping him shift his focus from parental conflict and management of the child's behavior to the improvement of the quality of his caregiving relationship with his child. Supporting his parenting capacities and self-reflection would help him recognize and respond more directly to his child's needs.

### Procedure

Pre- and post-intervention assessments were administered with M. before and 10 days after he completed the 8-week program. The clinician was a female psychologist-psychotherapist who completed the 4-day COS-P training and registered as a COS Parent Educator. The Italian version of COS-P program DVDs were used (COS-P Italian). With a pre/post-intervention design a series of measures were used to assess M.'s attachment status, parental behavior, parental perception of child's behavioral adjustment, and the level of co-parenting alliance. The performance-based tool and the self-report questionnaires were administered in two different sessions 1 week apart. The Adult Attachment Projective Picture System protocols were coded by two reliable judges qualified for coding.

### Measures

#### The adult attachment projective picture system (AAP; george and west, 2001)

The AAP measures adult attachment status based on the analysis of a set of projective stimuli. It consists of eight cards with line drawings on them: a neutral warm-up scene and then seven scenes depicting increasingly difficult attachment threats. The scenes represent children or adults alone (Alone pictures) or in potential attachment–caregiving relationships (Dyadic pictures). Subjects are asked to tell stories related to the pictures in which they have to describe what is going on in the picture, what led up to the scene, what characters are thinking or feeling and what might happen next. The stories are recorded, transcribed verbatim, and rigorously coded. Coding include seven dimensions that evaluate patterns of responses and the attachment content story. On basis of this set of dimensions, the protocols are classified into four attachment groups: Secure (F), Dismissing (Ds), Preoccupied (P), and Unresolved (U). The seven dimensions are included in three major categories: story content, discourse, and defensive process. The story content related to the hypothetical characters portrayed in the stimuli, included agency of self and connectedness for alone stories and synchrony for dyadic pictures (George and West, 2011). The agency of self is the capacity to maintain the self-integrity and to preserve organized thought and behavior in stressful contexts (e.g., solitude, death, isolation). There are three degrees to which the self moved psychologically or behaviorally toward the empowerment or integration: (1) exploring own IWMs, through descriptions of genuine self-reflection or thoughtfulness; (2) using the relationships to re-establish attachment equilibrium and to assuage or calm the attachment system; (3) engaging in behaviors that produces change (West and George, 2002). The connectedness assesses the desire and ability of the individual to be connected to the other in intimate attachment, friendship, or partnered relationships. The synchrony assesses the degree to which the individual is able to describe reciprocal interaction. The discourse codes evaluate the ability to maintain the self–other boundaries, and personal experiences indicate a loss of distance from the tables. The defensive processes, protecting from attachment stressful stimulus, are: deactivation, cognitive disconnection, and segregated systems. Deactivation process produces a distance between the individual and the attachment-activating event and is coded for story themes that underline the importance of rules, social scripts, power, achievement, and authority. The cognitive disconnection processes disconnect the elements of attachment from their source, thus undermining consistency and the capability of holding in one's mind a unitary view of events, emotions, and the individuals associated with them (George and West, 2011). Cognitive disconnection describes a form of defensive exclusion associated with uncertainty, ambivalence, and mental preoccupation with experiences, individuals, or feelings. Finally, segregated system describes a mental state in which painful attachment-related memories are isolated and blocked from conscious thought.

The AAP provides to researchers and clinicians with a construct-validated measure of attachment that preserves the emphasis on mental representation and defensive processes that is one of the primary features of attachment theory. The AAP has been validated using the Adult Attachment Interview (AAI; George et al., 1996) classification, with a convergent agreement between both for the four major attachments groups was 0.85 (kappa = 0.84, *p* < 0.001) (George and West, 2001, 2014). Studies have shown a strong inter-judge reliability of 0.86 (kappa = 0.79, *p* < 0.001). The AAP has shown a good test-retest reliability as George and West (2001) found that with a sample of 69 participants after 3 months 84% were classified in the same categories (kappa = 0.78, *p* < 0.001).

#### The parenting stress index-short form (PSI-SF; abidin, 1995)

The PSI-SF is a 36-item self-report measure of parenting stress in the parent/child relationship and consists of 3 subscales, each with 12 five- point Likert scale that ranges from 1 (strongly agree) to 5 (strongly disagree). The “Parent–Child Dysfunctional Interaction” subscale reflects parent/child relationship difficulties (range 12–60) (e.g., My child smiles at me much less than I expected). The “Parental Distress” subscale reflects parental stress experienced when raising own child (range 12–60) (e.g., There are quite a few things that bother me about life) and the “Difficult Child” subscale (range 12–60) (e.g., I think my child is very moody and easily upset). PSI-SF has been validated with studies showing reduction in parenting stress following parent training (Abidin, 1995), positive correlation with child disruptive behaviors (Ross et al., 1998), positive relationship with maternal depression (Webster-Stratton, 1988), and negative association with parents' sense of competency (McBride, 1989). PSI-SF internal consistency is high (α = 0.91), and test–retest reliability over a 6-month retest interval is good for the Total Stress score (*r* = 0.84) (Abidin, 1995). In this paper the Italian version was used and results compared with Italian normative data (Guarino et al., 2008).

#### The strengths and difficulties questionnaire (SDQ; goodman, 1997)

SDQ is a brief behavioral screening questionnaire for emotional, relational, and behavioral disorders in children and adolescents aged 4–16 years (Goodman, 1999; Goodman and Scott, 1999; Mathai et al., 2002). The SDQ consists of 5 groups of 5 items each (25 items total): emotional symptoms, conduct problems, hyperactivity/inattention, peer relationship problems, and pro-social behavior. Responses to each of the 25 items consisted of 3 options: not true, somewhat true, or certainly true. For all scales, negatively worded items are assigned scores of 2 for certainly true, 1 for somewhat true, and 0 for not true. Response to all other questions scored 0, 1, 2 so that a higher score indicated higher risk of emotional and behavioral problems. SDQ has been found to have good psychometric features both in clinical and non-clinical samples (Goodman and Scott, 1999; Goodman, 2001). The psychometric data indicated a mean internal consistency of 0.73 as measured with Chronbach's alpha (Goodman, 2001). The Italian version was used (Di Riso et al., 2010) and results compared with UK Normative data (Goodman, 2001) being Italian normative data for preschool children not available.

#### The parenting alliance measure (PAM; konold and abidin, 2001)

The Italian version of PAM scale was used to evaluate the co-parenting alliance (Camisasca et al., 2013). The PAM is a 20-item self-report instrument that assesses the strength of the perceived alliance between parents of children aged 1–19 years. This instrument assesses the parenting aspects of a couple's relationship (e.g., the levels of cooperation, communication, and mutual respect they exhibit with regard to their children's care). Parents respond to the items using a 5-point Likert scale that ranges from 1 (strongly disagree) to 5 (strongly agree), with a total possible range of 20–100, with higher scores indicating a stronger co-parenting alliance and greater reciprocity in the parental role. Previous studies have reported that PAM scores are positively correlated with marital quality and family functioning and negatively correlated with parenting stress and maladaptive family functioning (Feinberg et al., 2007). The PAM has demonstrated good psychometric characteristics with high degree of internal consistency (α = 0.97) and a good test-retest reliability (*r* = 0.80) (Konold and Abidin, 2001). Results were compared with USA normative data (Konold and Abidin, 2001) because Italian normative data are not available.

### Data analysis

The clinical significance analysis method proposed by Jacobson and Truax (1991) (“JT method”) for case studies using self-report measures from pre to post-intervention was used. The JT method proposes a two-step criterion: (a) by the end of therapy, client should change from a dysfunctional range to a functional range; and (b) the magnitude of the change should be statistically reliable (McGlinchey et al., 2002). In order to evaluate the statistically reliable magnitude of the change, the reliable change index (RCI) was used (Jacobson et al., 1984). The RCI is the change in a client's pre- and post- treatment score divided by the standard error of the difference for the test being used. The RCI shows whether clients changed sufficiently such that the change exceeds measurement error. If the RCI is 1.96 or greater, the change in scores because of treatment is statistically significant (1.96 equates to the 95% confidence interval). Based on the two criteria, the JT method classifies individual as: *recovered*, if both criteria are passed; *improved*, if the RCI but not the cut-off criteria is passed; *unchanged*, if neither criteria are passed; and *deteriorated*, if the RCI criteria is passed but the direction of change is toward dysfunction (Carlson et al., 2012).

## Results

### The adult attachment projective picture system (AAP; george and west, 2001)

General AAP attachment classification is discussed below. M.'s responses to some AAP pictures are then presented followed by a description of the story's unique feature of attachment interpreted using the AAP coding system. Pictures presented were selected as examples of significant changes in the underlying dimension of IWMs functioning.

### Attachment classification discussion

M.'s AAP pre and post were both coded and classified as insecure, specifically as Dismissing (Ds), because of his prominent use of the deactivation defense that allowed him to maintain distance in relationships. Stories often shifted attention away from distress to focus only on achievement or exploration. When distress was evident, he was quite able in describing what appeared on the surface, but the focus was typically superficial and not on the attachment relationship. Deactivation remained the principal way in which he minimized the influence of attachment stimuli, keeping strict self–other boundaries in relationships. M.'s modality to solve problems was modified from the exclusive use of action (agency = capacity to act) to the use of thoughtful and self-exploration to manage distress evoked to the attachment stimulus (i.e., internalized secure base). Some stories of the post intervention phase suggested that M. began to display sufficient personal resources to allow him to better mentalize internal conflicts and build stable, cohesive internal representations of attachment figures. These can be considered as signal of a potential change and constitutes a positive sign of his ability to utilize the intervention for self-reflection and personal growth. Attachment figures are portrayed generally as providing functional care, but the level of synchrony was little bit improved from pre to post intervention. In the first protocol, the characters were described in a functional relationship and there was no mutually reciprocal interaction. In the second protocol with the bed pictures, the characters were portrayed in a reciprocal and mutually engaging relationship. M. showed gradual improvements in his capacity to identify and respond to his own and other's attachment-related needs.

#### Bench

**Pre**: *A person … mh … a little bit … a little bit preoccupied … maybe she had to loose her home or she is preoccupied because she had received a bad break … relative's death or a economic loss or home's loss… I don't know if she is outside or if she is indoors … perhaps in a closed room, she refuses help … the bench is in a closed room and she stays there perhaps because she had a problem and she is waiting to be moved to another place more comfortable* (What might happen next?) *She could be better and she gets up and goes out* (Anything else?) *No*.

**Post**: *A woman staying on a bench in front of wall but… Initially it looks like a prison but then I see the grass and so I think that she outside in a garden. She sleeps and she thinks. She reflects and thinks about her life, about her projects, her difficulties, what she wants to do… then she can also sleep*. (What might happen next?). *She will wake up and she will go for a walk*. (Anything else?) *No*.

In the first story, the loneliness of the bench picture triggered segregated feelings of emptiness, isolation, and danger (lost, death). M. could only describe the segregated system and he was unable to envisage being connected to attachment figures or any other human beings at this moment. He demonstrated a capacity for reorganization through the girl's ability to make a decision, to take action and go out. This representational action designates a shift in the mindset that prevents him from becoming completely disorganized. In the post-intervention story, the bench picture stimulus has given the first indication of his ability to use solitude for self-exploration and inner reflection. Although he didn't articulate what the problem was about, the character in the scene was portrayed as using a self-reflective activity in an autonomous way, on his own initiative. Here M.'s change in managing the source of distress is noted as he shifted from a functional solution, to the use of a thoughtful and self-exploration solution.

#### Bed

**Pre:** *I see very clearly me and C. when she did not want to sleep and I stayed with her for a long time, I prepared all things in a comfortable way, for example the computer for the cartoons. I said “good night” to her and after a while she calls me and I stay close to her … to give her a hug and to caress her … It's a story of a boy that doesn't want to go bed. He needs his parent to be close to him because he is upset and he isn't relaxed… The parent tries to explains to him that it is time to sleep and then he gives him a hug … could be the scene before that the parent stays more close to his son or the scene when the son calls the parent or the scene after the hug when the child is ready to sleep… In reality I must go to my daughter again four times before she falls asleep … I get up, I get the glass of water and I take it to her and then she wants chocolate … this scene is very similar to what happen with C*. (Anything else?) *No*.

**Post:** *A mother with her child … the child asks her for a hug or to take himself in her arms. He is awake and with open arms. When he looks at his mother he wants to take her in his arms. She is a caring mother and she hugs her son*. (What are they thinking and feeling?) *The child wants to be hugged and the mother has a pain in her hand but she will be a good mother and be there*. (What might happen next?) *They prepare themselves and then go out*. (Anything else?) *No*.

In the pre-intervention story, material about the father's own experience was present. M. is being consciously reminded of events from his own life. The second story was free from M.'s personal experience as he was able to tell a story about hypothetical characters and events without autobiographical elements. In father's second stories, the mother responds promptly and appropriately to the child's need. This suggests a healthy capacity to appeal to attachment relationships for comfort, with the clear expectation that it will be provided. However, there was also an attempt to shift attention away from attachment events: the child's need of care was neutralized by deactivating defenses with a theme that emphasized the importance of a social rule (the mother has a duty to perform).

#### Cemetery

**Pre:** *He is a man that reads an inscription on the gravestone of his relative or friend or stranger … I'm not able to find a link between him and the gravestone but at the same time I think that there‘s neither a close parent nor a stranger… I think that it is a person known in books… it's a gravestone of an author, a historic character… The man remembers the cultural and philosophic message of this writer* (What might happen next?)*. Then the man returns home*. (Anything else?) *No*.

**Post:** *A man stands in front of a gravestone… he is a little bit sad and he remembers a friend or a particular person for him. It's a classic Sunday visit to the cemetery. The man thinks about the peculiarities of his friend and about the beautiful things they did together*. (What might happen next?) *and then he prays and returns home and he is satisfied with this moment*. (Anything else?) *No*.

In the two stories, this picture stimuli evokes in M. feelings of loss, a common theme, thus suggesting that the attachment conflict is recognized. In both stories, he attempted to neutralize feelings through deactivation by invoking concepts of social roles. In the post-intervention story, he referenced his IWMs of attachment by thinking about the deceased. The story regarded a deceased person with whom the character has had a relationship and who influenced the character in some way. The description involved genuine self-reflection and thoughtfulness. M. became more able to refer to internal state in which security and self-integrity are derived from his internalized relationship to the attachment figure.

### The parent stress index-short form (PSI-SF; abidin, 1995)

In the pre-intervention phase, M. experienced more stress than the average parent and scored in a clinical range (Total Stress Score = 95° percentile). His score indicated that he was very distressed in his parenting abilities and he felt that he was not able to respond to parenting tasks (Parental Distress = 90° percentile). He was also highly distressed by the quality of his relationship with his daughter. M.'s daughter didn't meet his expectations. M. felt alienated from his child and felt to some degree rejected by the child's behavior (Parent Child Dysfunctional Interaction = 90° percentile). He perceived his daughter's behavior as excessively disruptive or destructive to their relationship (Difficult Child = 95° percentile).

After participating in COS-P, using to JT method, M.'s results had significantly changed resulting recovered in parenting stress for the Parental Distress subscale and improved for the Total Score and Child Dysfunctional Interaction subscale. The scale for the Difficult Child subscale remained unchanged. In the post-intervention phase, M.'s level of parenting stress significantly improved and at this time, it fell in the borderline range (Total Stress Score = 84° percentile). He perceived that his lower distress was linked to his features as a parent and he felt that he was more able to manage his child's needs and requests with the score in the average range (Parental Distress = 60° percentile). In addition, significant improvement was observed in the father-child relationship. M. appeared more attuned with his child and their interaction and the dyad demonstrate increased mutual involvement (Parent Child Dysfunctional Interaction = 70° percentile). No changes in the perception of child problems was found (Difficult Child = 90° percentile) (Table 1).

**Table 1:** **Father's self reports scores pre- and post-intervention**.

**Measures**	**Pre-intervention**	**Post-intervention**	**RCI**
PSI-SF total stress score	101	84	≥1.96
PSI-SF parental distress	35	25	≥1.96
PSI-SF parent-child dysfunctional interaction	29	24	≥1.96
PSI-SF difficult child	37	35	<1.96
SDQ total score	19	10	≥1.96
SDQ emotional symptoms	2	1	<1.96
SDQ conduct problems	3	3	<1.96
SDQ hyperactivity/inattention	8	4	≥1.96
SDQ peer relationship problems	6	2	≥1.96
SDQ prosocial behavior	5	8	≥1.96
PAM	52	71	≥1.96

*PSI-SF, Parenting Stress Index-Short Form; SDQ, Strengths and Difficulties Questionnaire; PAM, Parenting Alliance Measure*.

### The strengths and difficulties questionnaire (SDQ; goodman, 1997)

The Total Difficulties Score indicated a high risk of clinically significant problems. Difficulties were in the behavioral and social areas. M. rated his child as having severe difficulties of attention, concentration, and hyperactivity problems. He also indicated that his daughter had problematic relationship with peers. M. described her as solitary, not having many friends and getting along better with adults than with other children. He observed a low level of pro-social behaviors in the daughter that included difficulties in helping others (both children and adults). After participation in the parenting program, M.'s profile ameliorated with respect to the assessment phase (Total Score = 10). The score was close to average and unlikely to be clinically significant. M. reported good behavioral and emotionally functioning for the daughter. Relevant changes were reported for hyperactive problems (Hyperactivity/Inattention = 4) and relationship with peers (Peer Relationship Problems = 2). He perceived improvement in his daughter's pro-social abilities (Prosocial behavior = 8): she was described as more able to be considerate and to share personal belongings with other children. Both the total score and the subscales resulted as recovered according to JT method (Table 1).

### The parenting alliance measure (PAM; konold and abidin, 2001)

In the pre-intervention phase, M. reported very low level of parenting alliance (6° percentile) and the score fell well within the clinical range. He showed a problematic perspective of being cooperative, communicative, and mutually respectful with regard to his child's caregiving. High levels of couple conflict have influenced M.'s capacity to cooperate with partner in meeting the needs of the child. In the post-intervention phase, the level of parenting alliances lightly ameliorate (17° percentile). However, remaining in the clinical range, it shifted from problematic to a marginal quality of parenting alliance. According to JT method, the father crossed only one of the two-step criterions, the RCI but not the cut-off criteria, and he resulted as improved in parenting alliance. M. showed changes in his capacity to provide support to his daughter's mother in her role as a parent and he was more respectful of her opinions and appeared more able to communicate with her about their child. He seemed to invest more in his child, to be more involved in parenthood and childrearing. M. was more able to cooperate with the child's mother by nurturing the developmental needs of the daughter (Table 1).

## Discussion

The research presented in this paper was designed to show a clinical application of COS-P in order to reflect on some specific therapeutic tasks and on some contexts and clinical indicators appropriate for its application. Whereas preliminary data on the efficacy of COS intervention has been published, no studies have yet investigated the efficacy of COS-P. From the overall research data of this single case study, it seems that COS-P worked making M. feel more competent as a parent in the management of his daughter's needs and in his interaction with her, thus moving psychologically in the direction of integration and understanding. Slight improvements were also present at the level of parenting alliance.

Specifically, with regard to the first aim on the parent's IWMs, M.'s attachment pattern was classified as Dismissing before and after the parenting program intervention. He showed he relied heavily on a deactivation form of defensive exclusion in telling the stories, thus producing a representational “distance” between himself and the attachment-activating event and neutralizing the attachment related distress (Lis et al., 2011; Delvecchio et al., 2013). When describing interpersonal themes M. often failed to represent a reciprocal and mutually engaging relationship. Previous studies on the efficacy of COS intervention reported significant increase in the proportions of securely attached children after parents' participation to the intervention (Marvin et al., 2002; Hoffman et al., 2006). No studies on the COS intervention has yet investigated changes in the parent's attachment model. Recently a study protocol was published in which it will assess parent's state of mind by a pre/post administration of the Adult Attachment Interview (Ramsauer et al., 2014). Even though the parent's attachment pattern didn't change, M. showed an important increase in the agency of self, defined as the degree to which the self is moving psychologically or behaviorally in the direction of integration or understanding (George and West, 2001). He moved from an initial lower level of an external manifestation of agency of self, called capacity to act, to an upper level called internalized secure base. This change indicates the passage from the tendency to engage in behavior in order to cope with attachment distress, to the capacity to draw upon internal resources to gain a sense of security and to use more self-reflection. Gaining an increase in agency of self can be considered a change in the mechanisms that are underpinning his attachment organization and thus showing the activation of a process that could lead to the acquisition of increased attachment's security (Fonagy and Target, 1997; Pazzagli, 2011). The capacity to use thoughtful and self exploration in response to stressful attachment situation constitutes one of the main purposes of the COS-P, that is to help parents have more reflective capacity on internal processes that guide problematic patterns of parent/child interactions.

With regard to the second aim concerning improvements expected in parenting behaviors, data showed changes, statistically classified as improved in parenting stress and as recovered in perception of the child's functioning. Results highlighted important ameliorations at the level of parenting stress perceived by M. In particular, the improvements in the parent domain are related to the perception of less stress in some functioning aspects like sense of competence (confidence in the ability to control child's behavior and knowledge of child development) and attachment (sense of closeness) (Abidin, 1995). For M., the source of stress appeared to be relevantly decreased: he seemed to perceive lower distress linked to his role as parent and to feel himself more able to manage his child needs and requests. Differently, he still reported some source of stress caused by some salient daughter characteristics that make it difficult for him to manage her. This data are in line with the results assessing changes in parent's perception of child positive and negative attributes before and after the parenting program. Although his perception of the daughter's difficulties remains high, he reported relevant changes in describing the child's behaviors, emotions and relationship difficulties. Relevant changes occurred not only in the reported reduction of the child's difficulties, but also in his increased perception of her strengths. M. also reported improvements in the daughter's prosocial behavior, depicting the child's behaviors as more considerate and available to the needs of others.

These results agreed with the aim of COS-P to provide parents with new ways to connect with their child by enhancing the caregivers' appropriate and sensitive responses to the child's emotions while developing the capacity to recognize their child's needs. At the beginning, M. perceived all the aspects assessed in parenting and child functioning as highly problematic. After participating in COS-P he seemed more balanced in his descriptions by reporting both challenging and functional aspects of their relationship. COS-P works on helping the caregiver understand the attachment's needs behind the child's behavior thus reducing the tendency to target negative attributions of the child. Positive changes in parenting behaviors goes in the expected direction of COS-P. Research has showed significant associations between caregiver's experience of parenting stress while performing parenting responsibilities and increased risk of child maltreatment and, ultimately, the development of childhood psychological and behavioral disorders (Abidin, 1992; Abidin and Jenkins, 1992). Furthermore, the capacity to recognize aspects of strength in the daughter shows that the COS model works through valuing participants and highlighting positive child/parent interactions.

Finally, the third aim concerned an expected improvement in parental alliance. This change is consistent with previous research that suggests increased alliance is associated with the quality of parenting. Parental alliance refers to the quality of the alliance between the two parents with regards to communication and teamwork (Konold and Abidin, 2001). After participating in COS-P, M. reported a change in the co-parenting relationship from problematic to one with marginal quality. This improvement was statistically significant. For Zeanah et al. (2011) high levels of parental conflict increase the likelihood of attachment disturbances in children, perhaps through a reduced sensitivity in parents who are worried about their own concerns. In this case study, the amelioration of the parental alliance could explain M.'s greater capacity to recognize his daughter's attachment needs. The narratives of father's AAP also showed enhanced recognition of the child's needs for proximity and care from both parents. To the table called “bed,” showing a baby on bed that stretches out his hand to a stylized female figure, at the beginning M. narrowed his difficult experience with the daughter when going to sleep, after the participation to COS-P he depicted a mother-child interaction, without showing the need to replace himself to the female figure. Higher strength of childrearing alliance between parents provides an improved attachment outcome for child at risk for insecure attachment (Castellano et al., 2013).

The overall improvements showed by this research data agreed with M.'s comments of satisfaction and appreciation with participation in the COS-P. He described the experience as highly positive with particular emphasis on two aspects. M. declared that, despite having always imagined that his childhood history would have an influence in his life in general and his relationship with the daughter in particular, COS-P helped him to identify problematic areas and specific mechanisms related to those influences, allowing him to be more aware of the quality and characteristics of the relationship with his daughter and thus helping him to implement reparative behaviors. Another element that really struck M. was related to working through the parental role characteristics (being always bigger stronger, wiser and kind, to content needs when possible, and take charge when necessary). His reflection on his role allowed him to more clearly identify the times in which he pushed his daughter to provide care for him and seek her support and reinforcement. After the intervention, he described his relationship with his daughter as not only having some difficulties but also as having more moments of enjoyment and sharing. Furthermore, he started thinking about beginning an individual psychotherapy.

## Conclusion

The present study is the first to show a clinical application of COS-P. A single case-study was presented of a father's treatment in a high parental conflict levels. As pointed out by Zeanah et al. (2011) in similar situations attachment perspectives should have a central role in interventions aimed at restoring interpersonal connections and establishing a safe and secure living situation for the child. M.'s initial motivation was characterized by a low will to work on relational struggles and parenting functions through a psychotherapy treatment. Instead it seemed that M.'s motivation was mostly characterized by extrinsic factors instrumental to the achievement of a pre-established outcome (e.g., seek revenge with the child's mother via applying for daughter's custody). After COS-P participation, father/child's mother antagonism diminished as they became more cooperative, conflict for child custody decreased as the judicial conflict on daughter's custody eclipsed.

The COS-P, differs from other visually based attachment approaches, as it uses materials and archived videotapes of parent/child interactions selected as examples of core COS model components without the aid of “individualized” video-feedback. For these reasons, COS-P has the advantage of being more flexible in terms of timing and practicality. In the case study presented, empirical data and clinical issues showed a trend toward increased capacity for integration and understanding in term of agency of self, along with a increased capacity to recognize the child's needs. Participation in COS-P allows participants to face personal relational struggles in a less direct and immediate way with respect to more individualized interventions. The intervention activated a process of involvement and self reflection that led to an increased capacity to recognize M.'s personal relational dynamic role as well as an increased motivation to pursue a deeper personal psychotherapy. A difficult-to-reach caregiver with low intrinsic motivation was an appropriate candidate for the application of COS-P.

The current study adds to the literature on attachment-based interventions by providing preliminary data from a single case-study on the efficacy of COS-P. The article concerned a single case pilot study and this has implications for the generalizability of the results. Yet the fact that it involved a single case study, didn't evaluate the process therapeutic alliance between therapist and caregiver, and didn't assess the father/child's interaction by systematic observations are three core limitations. Randomized controlled trial on COS-P are needed in order to expand the preliminary research data on COS-P and to better individualize the clinical indications and contexts appropriate for its application.

## Author contributions

Chiara Pazzagli: Substantial contributions to the conception and design of the work and to the interpretation of data; Drafting the work; Final approval of the version to be published; Agreement to be accountable for all aspects of the work. Loredana Laghezza: Substantial contributions to the design of the work and to the analysis of data; Drafting the work; Final approval of the version to be published; Agreement to be accountable for all aspects of the work. Francesca Manaresi: Acquisition of data for the work; Revising the work critically; Final approval of the version to be published; Agreement to be accountable for all aspects of the work. Claudia Mazzeschi: Substantial contributions to the design of the work and interpretation of data; Revising the work critically; Final approval of the version to be published; Agreement to be accountable for all aspects of the work. Bert Powell: Substantial contributions to the conception of the work; Revising the work critically; Final approval of the version to be published; Agreement to be accountable for all aspects of the work.

### Conflict of interest statement

The authors declare that the research was conducted in the absence of any commercial or financial relationships that could be construed as a potential conflict of interest.
